# Supplementary material for: Mitigation of Arctic permafrost carbon loss through stratospheric aerosol geoengineering
Source: Nat Commun. 2020 May 15;11:2430. doi: 10.1038/s41467-020-16357-8 (PMC7229154; doi:10.1038/s41467-020-16357-8)
Supplement: Supplementary file 1 — Supplementary Information [file 41467_2020_16357_MOESM1_ESM.pdf]

Supplementary Information for:

Mitigation of Arctic permafrost carbon loss through stratospheric aerosol geoengineering

Chen et al., Nature Communications

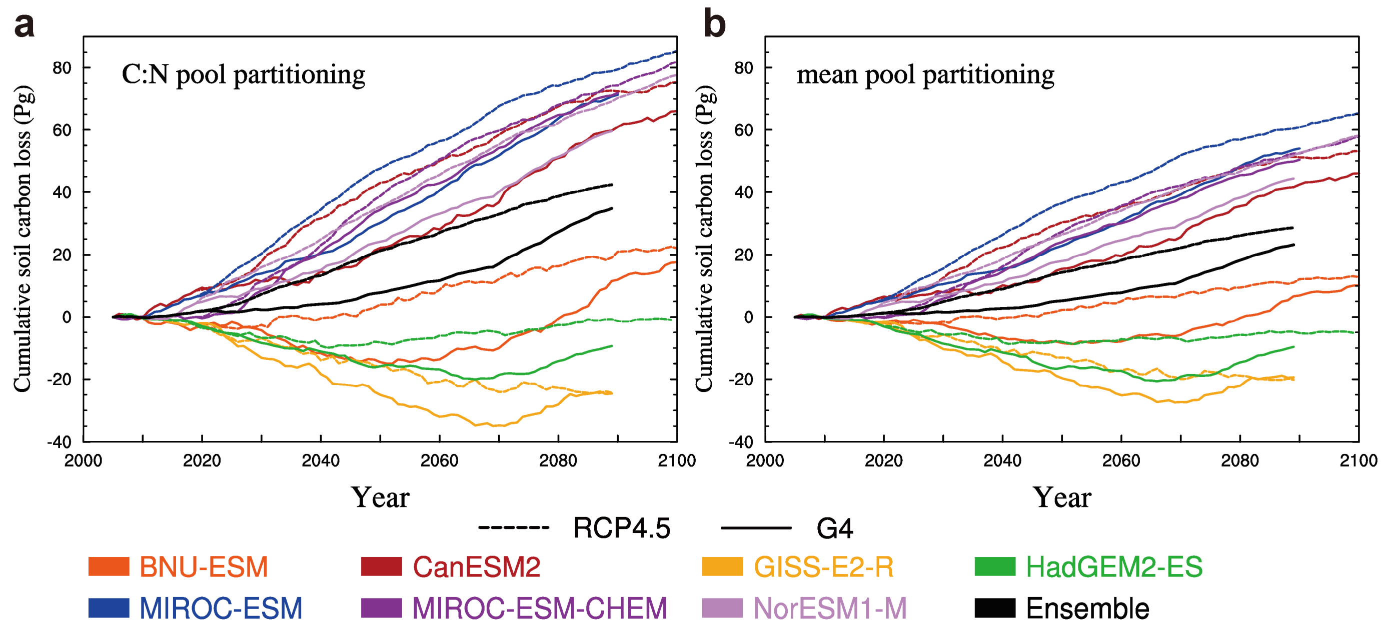

Supplementary Figure 1. Simulated soil C loss of two pool partitioning methods. Cumulative losses of permafrost C storage between 2006 and 2100 under the emission scenario RCP4.5 and the geoengineering experiment G4, simulated by PInc-PanTher in two pool partition ways. The pool partitioning method determines the proportion between the parallel three-pool of permafrost soils (fast, slow, and passive pools), and thus determines the rate of soil carbon decomposition. The C:N ratio method derives a regression relationship between the fraction of C in pools with different turnover times and soil C:N ratio. The mean pool partitioning method determines decomposition parameters for organic and mineral horizons separately, where organic horizons are defined as those having more than 20% C by mass and mineral horizons are those that have less than 20% C. The simulated soil C loss of mean pool partitioning method is consistently about 70–80% as large as the C:N pool partitioning method.

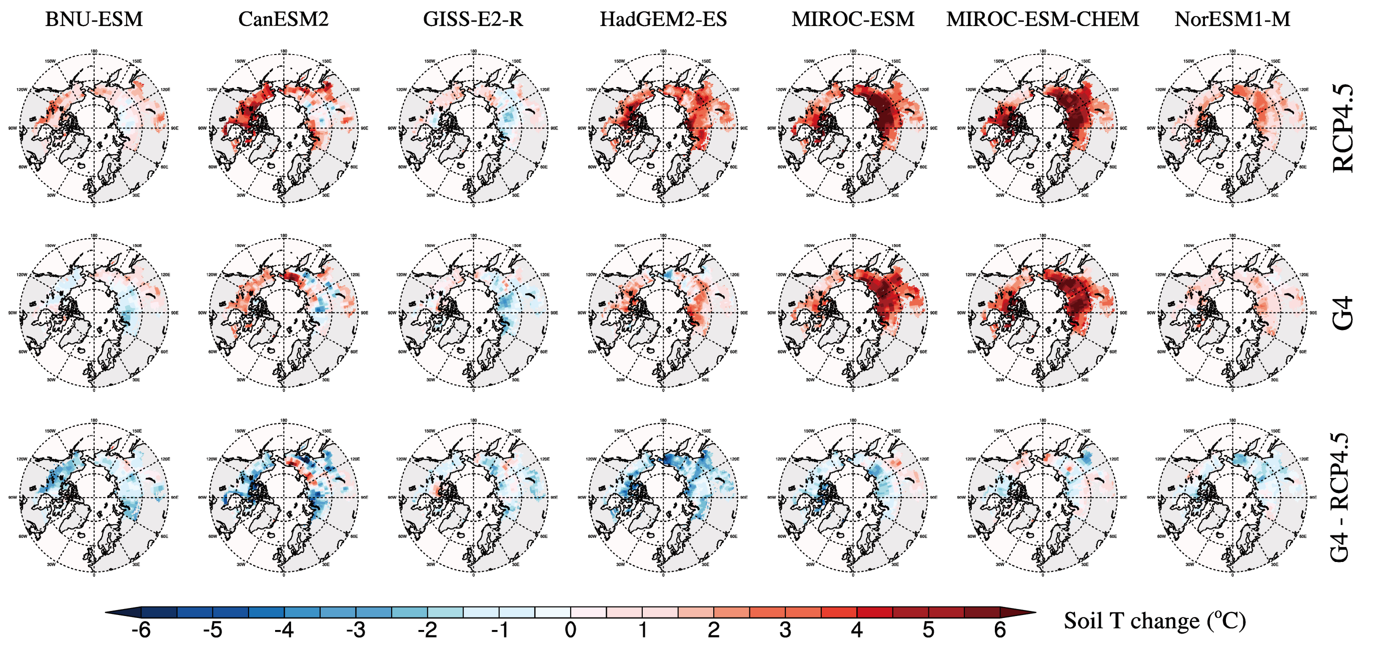

Supplementary Figure 2. Projected soil temperature change between 2020 and 2069. Maps of soil T change between 2020 and 2069 under the emission scenario RCP4.5 and the geoengineering experiment G4, as well as the difference (G4-RCP4.5), averaged over the upper 3 meters. The relative soil T changes are (1.3±1.2 °C) under G4, (2.2±1.2 °C) under RCP4.5 and (-0.9±0.4 °C) for G4-RCP4.5. The cooling effect of geoengineering is well-replicated among all ESMs that have run G4 experiment.

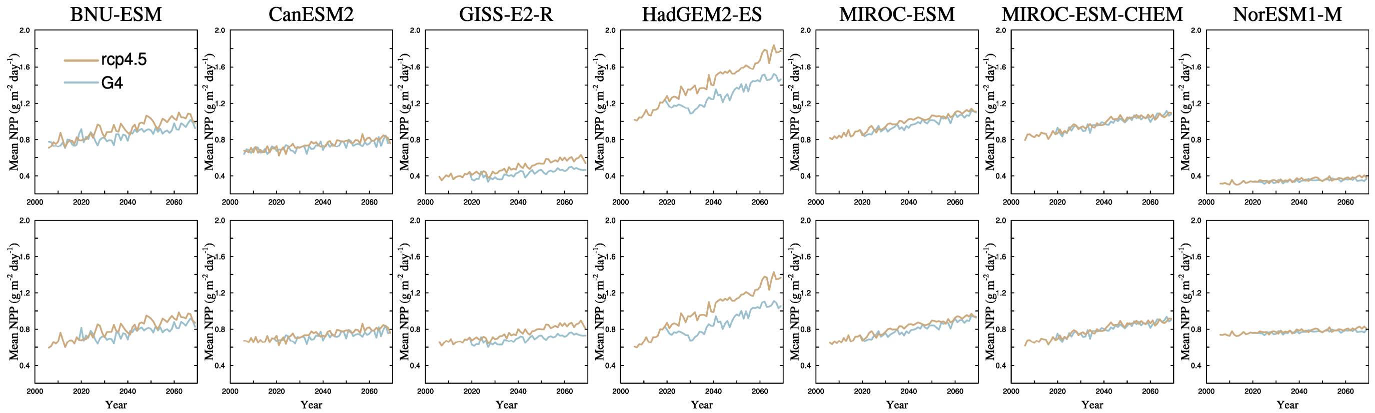

Supplementary Figure 3. Bias-correction of net primary productivity. NPP in permafrost region before (top) and after (bottom) bias correction for RCP4.5 (orange) and G4 (blue). The NPP outputs of ESMs are bias-corrected by the continuous and widely used MODIS annual NPP products.

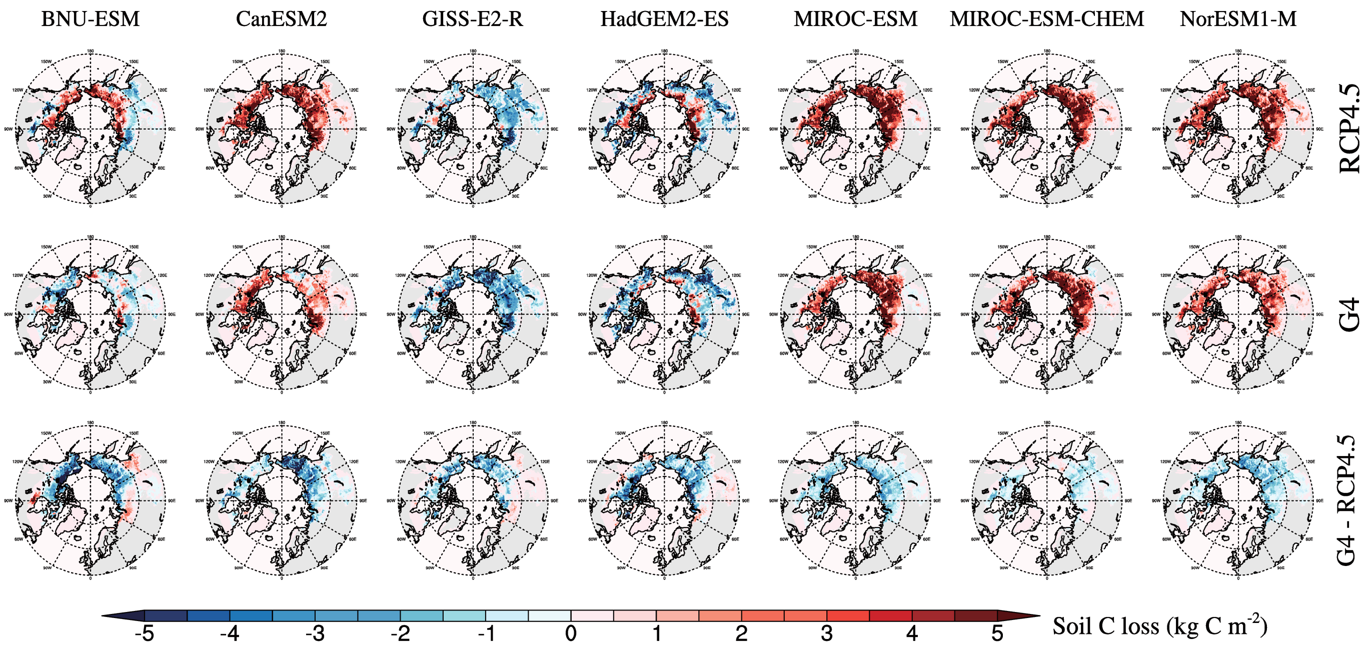

Supplementary Figure 4. Projected soil carbon loss between 2020 and 2069. Maps of permafrost soil C losses between 2020 and 2069 under the emission scenario RCP4.5 and the geoengineering experiment G4, as well as the difference (G4-RCP4.5), calculated by PInc-Panther. MIROC-ESM and MIROC-ESM-CHEM, with simulations of warming above 3°C, produce severe soil C losses, while GISS-E2-R with minor soil temperature change produces net soil C gains under both scenarios before 2070.

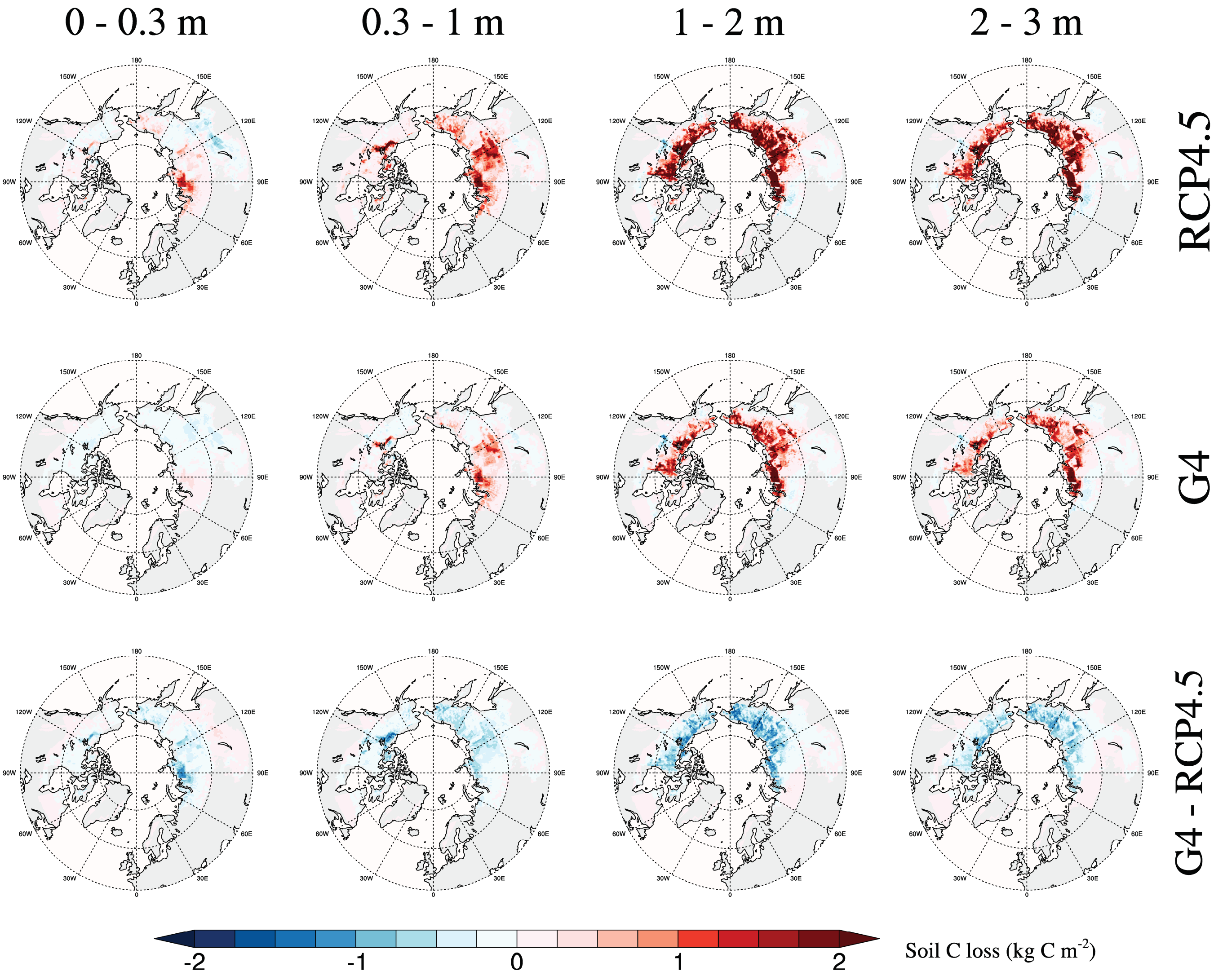

Supplementary Figure 5. Permafrost soil carbon losses at different depth. Maps of permafrost soil C losses of the ensemble value between 2020 and 2069 under the emission scenario RCP4.5 and the geoengineering experiment G4, as well as the difference (G4-RCP4.5), for four depths: 0-0.3m, 0.3-1m, 1-2m and 2-3m. In the four layers permafrost C loss under the G4 scenario are -1.0, 2.0, 6.1 and 4.8 Pg respectively, and 0.7, 5.8, 10.7 and 8.4 Pg for the RCP4.5 scenario. Soil C loss in the upper meter are reduced by ~85% relative to RCP4.5, while the G4 experiment mitigates about 40% of soil C losses in the deeper soils (2-3 m).

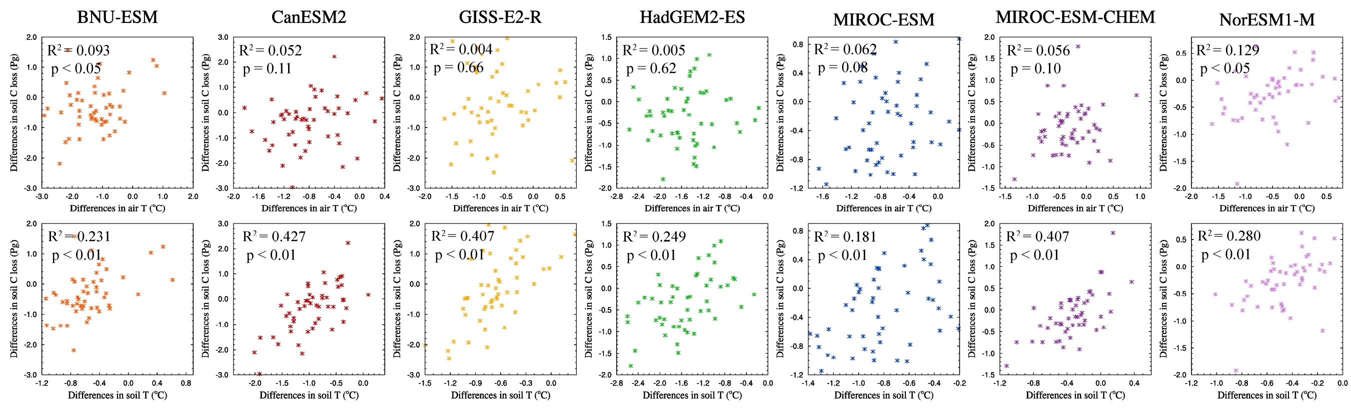

Supplementary Figure 6. Relationships between soil C loss and both soil and air T. Scatterplots of the yearly (2020 to 2069) differences in soil carbon loss (G4-RCP4.5) as a function of surface temperature differences (top row) and soil temperature (TSL) differences (bottom row). The R^2 and p values of the linear regressions are shown on each panel. TSL is a far better predictor of soil C losses than surface temperature forcing, but is still far from sufficient to provide the soil C balance prediction.

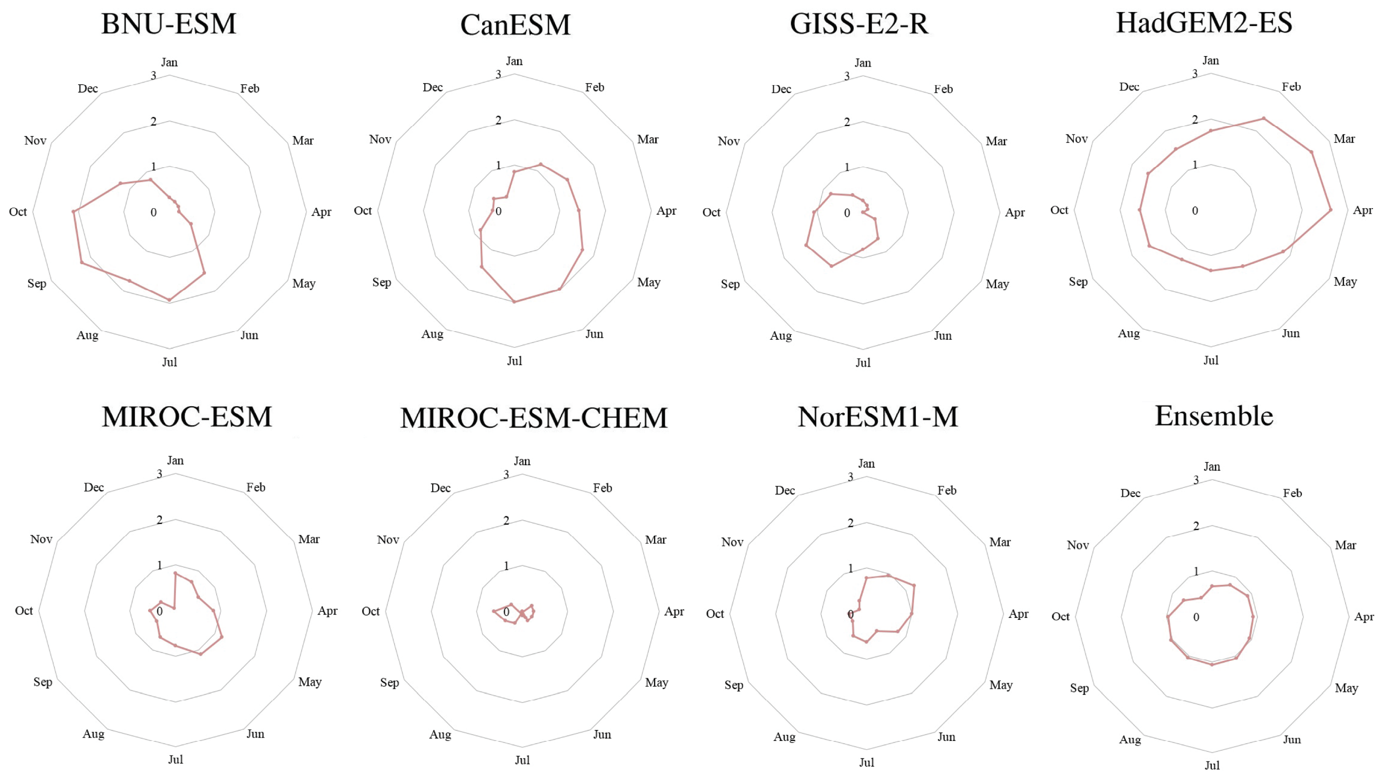

Supplementary Figure 7. The monthly cooling effect of G4 relative to RCP4.5. Monthly differences (G4-RCP4.5; units:°C) in soil temperature for each earth system model over the 2020-2069 period. Soil temperature reduced by the geoengineering experiment G4 are most for the HadGEM2-ES simulations and least for MIROC-ESM-CHEM, while the ensemble mean value shows that the cooling effect of G4 relative to RCP4.5 remains at about 1 degree Celsius from January to October.

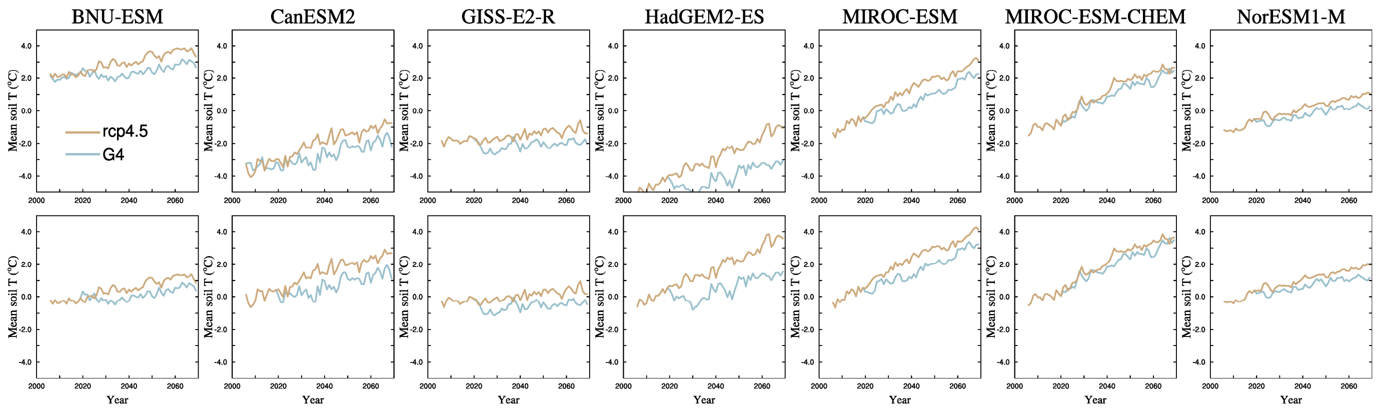

Supplementary Figure 8. Bias-correction of soil temperature. Mean annual soil temperature in permafrost region before (top) and after (bottom) bias correction for the emission scenario RCP4.5 (orange) and the geoengineering experiment G4 (blue). Soil and snow physical processes that determine the thermal properties of permafrost soils are poorly represented in the CMIP5 generation of earth system models, so we perform the initial bias-correction for better simulations.

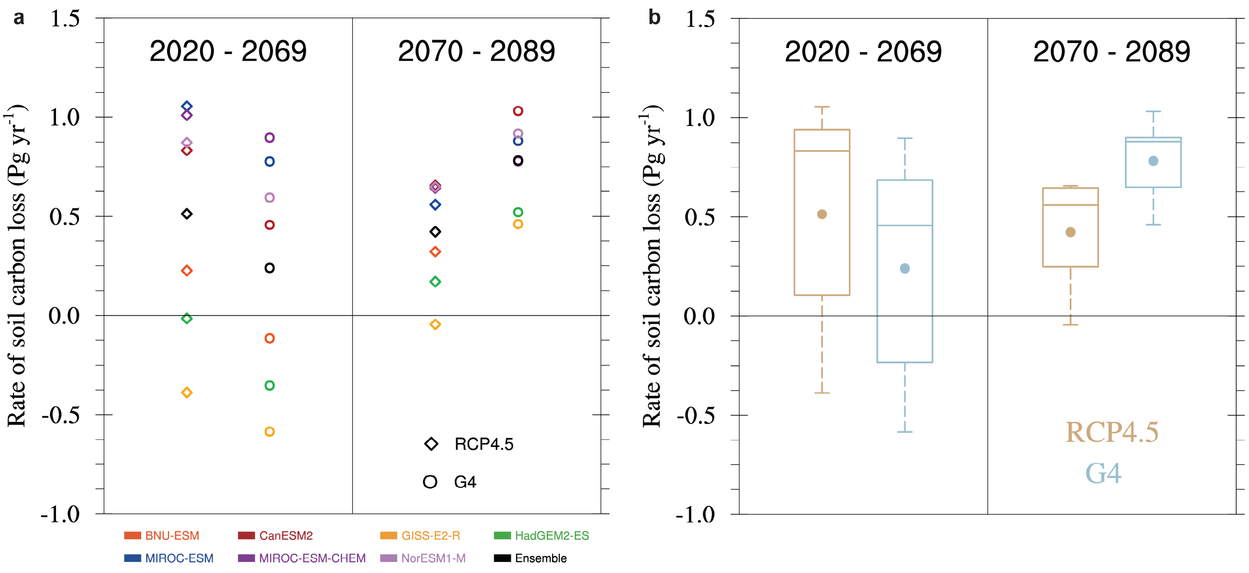

Supplementary Figure 9. Rate of soil carbon loss and the termination shock. (**a**) Dot plot of integrated permafrost C loss under the emission scenario RCP4.5 and the geoengineering experiment G4. (**b**) box-plot of integrated permafrost C loss. Whiskers: minimum and maximum values; boxes: 25–75% range; horizontal lines: median; dots: ensemble mean. The rise in surface air temperatures following termination causes a strong air-soil temperature gradient that drives heat into the soil generating a sudden increase in permafrost emissions from 2070-2089 which partially offsets the carbon loses avoided during the G4 implementation.

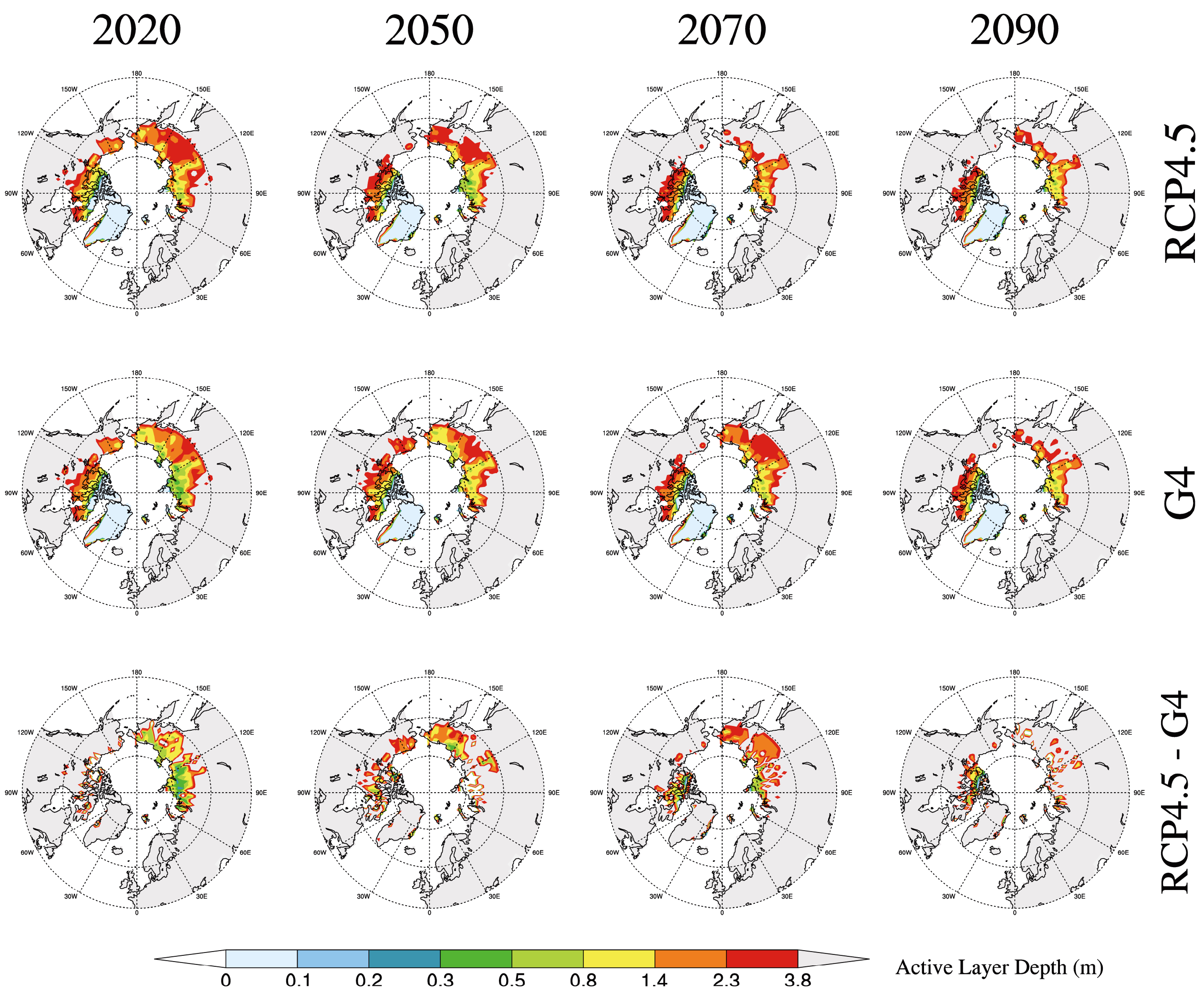

Supplementary Figure 10. Simulation of permafrost extent and active layer depth. Modeled near-surface permafrost extent and active layer depth under the emission scenario RCP4.5 and the geoengineering experiment G4, calculated by the NorESM1-M soil temperature. Gray denotes area where has no permafrost or the active layer depth is greater than 3.8 m.

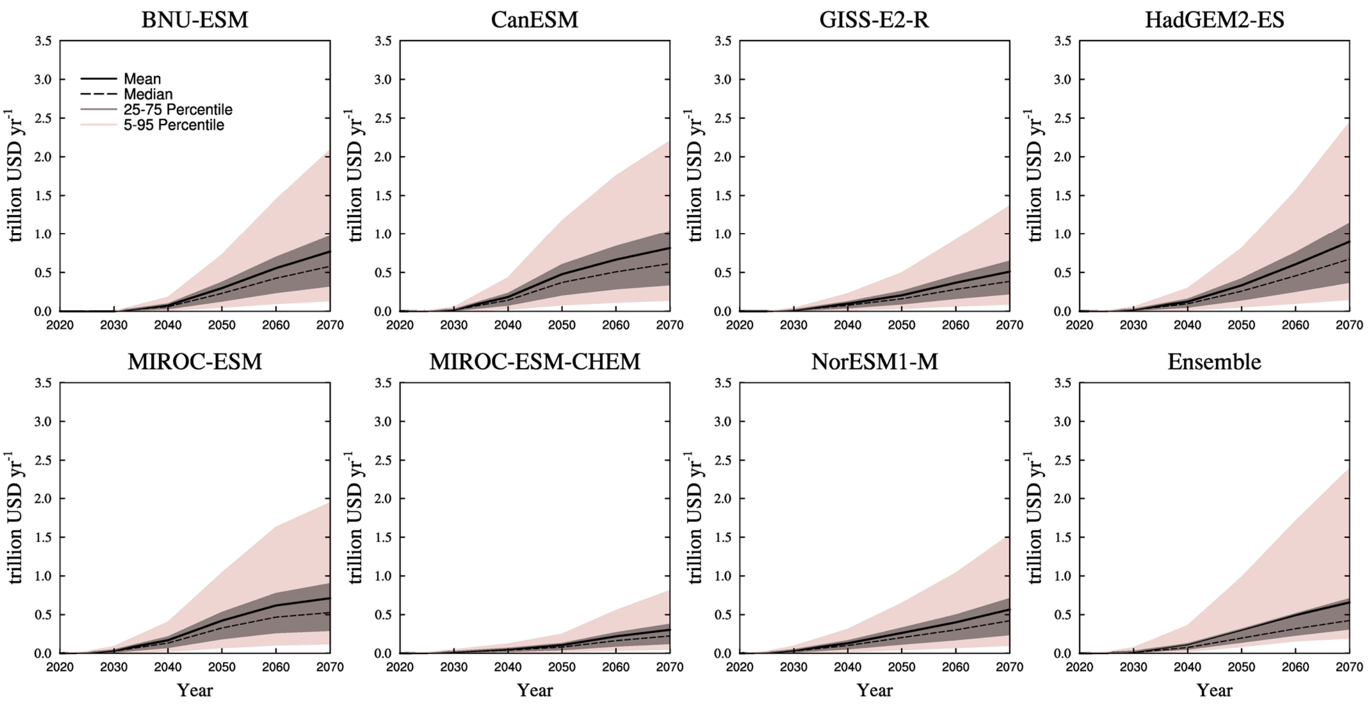

Supplementary Figure 11. Economic benefits from the mitigation of permafrost carbon loss. The difference between RCP4.5 and G4 scenarios (RCP4.5 - G4) of predicted annual economic impacts due to permafrost carbon–climate feedbacks, obtained from 100,000 Monte-Carlo runs of PAGE-ICE driven by projected permafrost CO_2 and CH_4 emissions. Solid black lines represent the mean values; dotted black lines show the median values; light and dark color intervals show 5–95% and 25–75% confidence intervals, respectively. Net economic losses reduced by SAI are most for the CanESM2 simulations and least for MIROC-ESM-CHEM, consistent with the ESMs estimates of mitigating permafrost C loss. All simulations report positive value with mean of US$0.6 trillion/yr and 5-95% range of US$ 0.2–2.4 trillion/yr in 2069, indicating great economic benefits of avoided PCF.

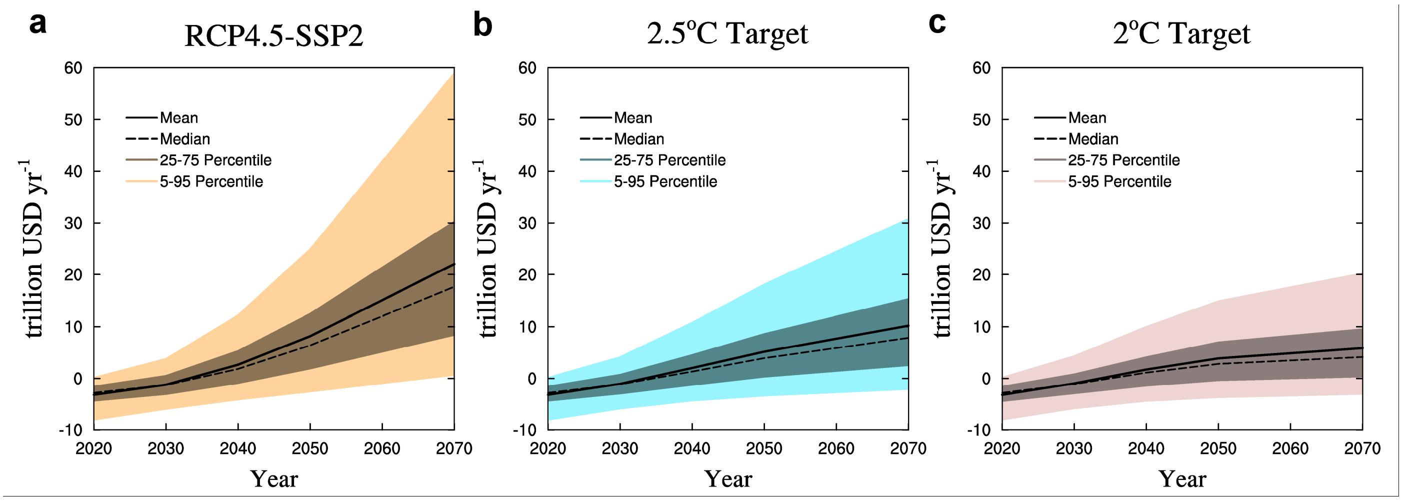

Supplementary Figure 12. Economic impacts of future climate change. Global economic impacts of climate change under three scenarios: RCP4.5-SSP2, the 2.5°C and 2°C targets, obtained from 100,000 Monte-Carlo runs of PAGE-ICE model driven by projected anthropogenic greenhouse gas emissions. In 2069, the economic losses due to climate change under RCP4.5, 2.5°C and 2°C targets will be US$ 22, 10 and 6 trillion per year, respectively.

Supplementary Table 1. Mitigation of permafrost soil carbon loss and CH_4 emissions. Differences in PInc-PanTher estimates of permafrost soil C loss (units: Pg) and CH_4 emissions (units: Tg) between G4 and RCP4.5 scenarios (G4 minus RCP4.5) during three phases: implementation phase (2020-2069), termination phase (2070-2089) and the whole experiment (2020-2089). Bold fonts are significant at 95 % level according to the Wilcoxon signed-rank test.

Soil C loss	BNU-ESM	CanESM2	GISS-E2-R	HadGEM2-ES	MIROC-ESM	MIROC-ESM-CHEM	NorESM1-M	Ensemble
2020-2069	**-17.1**	**-18.8**	-9.8	**-16.9**	**-13.9**	-5.6	**-13.9**	**-13.7**
2070-2089	**11.1**	**7.5**	**10.1**	**7.0**	**6.4**	2.7	**5.4**	**7.2**
2020-2089	-5.9	-11.3	0.3	-9.9	-7.4	-2.9	**-8.5**	**-6.5**
CH_4 emissions	BNU-ESM	CanESM2	GISS-E2-R	HadGEM2-ES	MIROC-ESM	MIROC-ESM-CHEM	NorESM1-M	Ensemble
2020-2069	**-269**	**-221**	**-330**	**-844**	**-219**	**-83**	**-116**	**-298**
2070-2089	-21	2	**-129**	**-145**	22	**40**	-2	**-34**
2020-2089	**-289**	**-219**	**-459**	**-989**	**-197**	-43	**-118**	**-332**

Supplementary Table 2. The impacts of geoengineering on net biological productivity. Differences in net biological productivity (NBP) between G4 and RCP4.5 scenarios (G4-RCP4.5, units: Pg) during 2020-2069 for 6 ESMs (GISS-E2-R does not report NBP). Positive values indicate more biological land carbon under G4 than RCP4.5. Bold fonts are significant at 95 % level according to the Wilcoxon signed-rank test. The NBP of the permafrost region varies across ESMs and accounts for only about 5% of the global NBP differences between scenarios, which is dominated by what happens in the tropics.

NBP fluxes	BNU-ESM	CanESM2	HadGEM2-ES	MIROC-ESM	MIROC-ESM-CHEM	NorESM1-M	Ensemble
Permafrost regions	1.92	1.17	**-18.47**	**16.87**	6.86	0.61	1.49
Tropics	42.12	**41.05**	**41.07**	16.65	-5.56	-2.72	**22.10**
Global	**69.94**	**51.8**	**42.86**	**43.24**	6.54	1.82	**36.03**
